# Walking in orthostatic tremor modulates tremor features and is characterized by impaired gait stability

**DOI:** 10.1038/s41598-018-32526-8

**Published:** 2018-09-20

**Authors:** M. Wuehr, C. Schlick, K. Möhwald, R. Schniepp

**Affiliations:** 1 German Center for Vertigo and Balance Disorders, University Hospital, LMU Munich, Munich, Germany; 2 Department of Neurology, University Hospital, LMU Munich, Munich, Germany

## Abstract

Primary orthostatic tremor (OT) is characterized by high-frequency lower-limb muscle contractions and a disabling sense of unsteadiness while standing. Patients consistently report a relief of symptoms when starting to ambulate. Here, we systematically examined and linked tremor and gait characteristics in patients with OT. Tremor and gait features were examined in nine OT patients and controls on a pressure-sensitive treadmill for one minute of walking framed by two one-minute periods of standing. Tremor characteristics were assessed by time-frequency analysis of surface EMG-recordings from four leg muscles. High-frequency tremor during standing (15.29 ± 0.17 Hz) persisted while walking but was consistently reset to higher frequencies (16.34 ± 0.25 Hz; p < 0.001). Tremor intensity was phase-dependently modulated, being predominantly observable during stance phases (p < 0.001). Tremor intensity scaled with the force applied during stepping (p < 0.001) and was linked to specific gait alterations, i.e., wide base walking (p = 0.019) and increased stride-to-stride fluctuations (p = 0.002). OT during walking persists but is reset to higher frequencies, indicating the involvement of supraspinal locomotor centers in the generation of OT rhythm. Tremor intensity is modulated during the gait cycle, pointing at specific pathways mediating the peripheral manifestation of OT. Finally, OT during walking is linked to gait alterations resembling a cerebellar and/or sensory ataxic gait disorder.

## Introduction

Primary orthostatic tremor (OT) is a unique clinical syndrome of unknown prevalence, characterized by a high-frequency pattern of coherent muscle contractions (13–18 Hz) in the lower limbs and trunk while standing^1–3^. The tremor is associated to the patient’s intense sense of unsteadiness and dizziness. The exact pathophysiological mechanisms underlying primary OT remain unresolved. However, a central oscillatory ponto-cerebello-thalamo-cortical network has recently been identified in patients with OT^4,5^.

Sitting or lying in OT typically lead to a complete relief of tremor activity and accompanying symptoms. During walking, most patients do not experience problems and report a profound relief of unsteadiness. However, patients in more advanced stages of the disease may face difficulties with tandem walking, walking slowly, or climbing stairs^6^. Furthermore, anecdotal reports in single patients with OT indicate that tremor activity may persist during walking^3,7,8^. However, it is hitherto unknown whether the ongoing tremor activity during ambulation is akin to that of standing or may exhibit specific changes with respect to frequency, intensity or coherence. It is further unclear whether OT during walking is linked to a specific gait disorder as observed in other types of tremor such as essential tremor^9^. Finally, it has to be resolved, why despite continuation of OT during walking, most patients consistently experience a substantial relief of subjective unsteadiness associated with activity.

To elucidate these questions, the present study systematically investigated the tremor activity and walking behavior of patients with OT. Tremor characteristics such as frequency, intensity, and coherence were evaluated by EMG-recordings during stance-walk and walk-stance transitions as well as for continuous steady-state walking. These findings were then linked to the walking performance of patients by simultaneous recordings of their spatiotemporal gait patterns.

## Methods

### Standard protocol approvals, registrations, and patients consent

The study protocol has been approved by the Ethics Committee of the University of Munich and was registered (DRKS00012907). All procedures were in accordance with the Helsinki declaration and patients gave their written informed consent.

### Subjects

Nine patients with primary OT (mean age 68.3 ± 8.7 years, four females) and nine healthy controls (mean age 66.2 ± 4.8 years, four females) participated in the study (detailed patient characteristics are presented in Table 1). Definite diagnosis of OT was made by surface EMG-recording exhibiting a coherent tremor between homologous leg muscles within a frequency range of 13–18 Hz. Patients underwent a standardized neurological examination to exclude additional signs indicative of a secondary OT (i.e., hypokinesia, rigidity, dystonia, failure of gait initiation).

**Table 1:** Patient characteristics including neurological findings and medication.

Patient	Sex	Age (years)	Tremor frequency (Hz)	Duration (years)	Medication	Neurological findings
1	m	62	14.5	1	no	1, 4
2	f	55	14	5	Gabapentin 1200 mg/d	1
3	m	75	17.5	10	no	1, 2, 4
4	m	75	14	14	Clonazepam 1 mg/d	1, 2, 3, 4
5	f	56	17	5	no	1
6	f	75	16.5	6	no	1, 2, 3, 4
7	f	66	14	13	no	1, 2, 4
8	m	75	13.5	4	Gabapentin 600 mg/d	1, 4
9	m	76	15	3	Levodopa/Benserazid 300/75 mg/d	1

f = female; m = male; neurological findings: 1 = postural instability, 2 = dysmetria, dysdiadochokinesia or intention tremor upper limbs (uni- or bilateral), 3 = dysmetria lower limbs (uni- or bilateral), 4 = diminished ankle reflexes and/or reduced vibration sense.

### Procedures

Walking performance of patients and controls was examined for 60 s at slow (0.42 m/s) and medium (0.84 m/s) gait speed on a pressure-sensitive treadmill (Zebris®, Isny, Germany; h/p/cosmos®, Nussdorf-Traunstein, Germany; 1.6 m long with a sampling rate of 100 Hz) in a randomized order^10^. Both walking trials were preceded and followed by 60 s periods of standing. Acceleration and deceleration periods to reach continuous walking speed or rest position had a duration of 1.2 s for slow and 2.0 s for fast speed, resulting in a total trial duration of 182.4 s and 184.0 s respectively. Each trial was started after an initial 15 s period of standing, i.e., when the high-frequency pattern of leg muscle contractions became visible in all patients.

Surface EMG activity during standing and walking in patients and controls was recorded with Ag/AgCl electrodes simultaneously from the tibialis anterior, gastrocnemius, biceps femoris and vastus medialis muscles of the dominant leg side using a Zebris DAB-Bluetooth device (Zebris®, Isny, Germany) at 1000 Hz. EMG signals were amplified, bandpass-filtered at 10–100 Hz and full-wave rectified. To reduce the inter-individual variability of the EMG recordings, EMG signals were further normalized to the peak EMG from the respective muscle during the two walking trials^11^.

### Data analysis

Tremor intensity and coherence between every combination of recorded muscle pairs were analyzed in three steps: (1) The average tremor characteristics were analyzed for the two standing and the in-between walking periods separately by spectral analysis using finite fast Fourier transform with a block size set to 2 s resulting in a frequency resolution of 0.5 Hz^12^. Furthermore, the average EMG level (aEMG) for the stance and walk periods was obtained by dividing the integrated EMG signal by the respective period duration^13^. (2) The time-dependent tremor behavior for the whole trial duration was assessed by time-frequency spectral analysis using short-time Fourier transform with a window length of 2 s overlapping by 0.05 s resulting in a frequency resolution of 0.5 Hz^14^. Time-dependent estimates of tremor intensity were used to calculate the tremor onset latency, i.e., the time required for the tremor after walking to regain the average intensity of the initial stance period. (3) Short-time Fourier transform was further used to examine the phase-dependent tremor behavior during the gait cycle according to a previously described procedure^15^: First, EMG signals were cut into strides synchronized to the right heel strike. Subsequently, short-time Fourier transform was used to compute the time-dependent auto- and cross-spectra for each stride cycle. To account for stride-to-stride variability, single-cycle auto- and cross-spectra were resampled to the average stride duration of the walking trial. Finally, power spectrum and coherence estimates were obtained by averaging across all stride cycles. The peak tremor intensity and coherence were calculated for the stance and swing phase of the gait cycle.

Walking performance for each trial was assessed by calculating the following gait cycle parameters: The mean and the coefficient of variation (CV) of stride length, stride time, and base of support as well as the percentage of swing and double support duration with respect to the total stride duration. The average force applied during walking was further assessed by dividing the integrated vertical ground reaction force (vGRF) by the total walking period duration. The average vGRF was further normalized to the individual body weight (BW) and expressed in %BW^16^.

### Statistical analysis

Data are reported as mean ± SEM. Activity effects on tremor frequency, intensity, and coherence were analyzed by repeated-measures analysis of covariance and Bonferroni post hoc analysis with group (OT vs. controls), gait speed (slow vs. medium), activity (standing vs. walking), and muscle type as factors and medication (yes vs. none) as covariate. Gait cycle phase effects on tremor intensity and coherence were analyzed correspondingly with gait speed, gait cycle phase (stance vs. swing period), and muscle type as factors and medication as covariate. Finally, alterations in gait performance were analyzed accordingly with group and gait speed as factors and medication as covariate. Pearson’s correlations were performed to assess possible significant relations between on the one hand tremor characteristics during walking and on the other hand gait cycle parameters as well as vGRF and aEMG levels during walking. Results were considered significant at *p* < 0.05.

## Results

**Figure 1:**
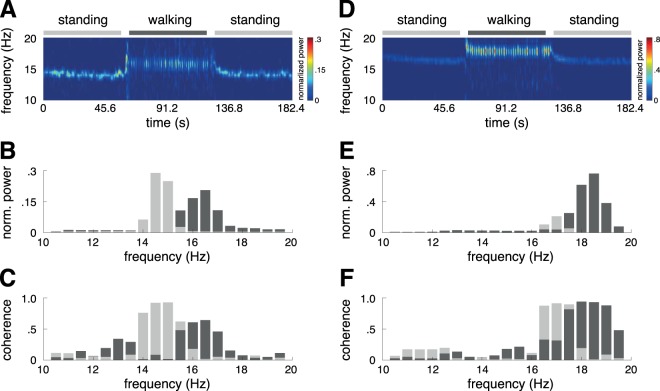
Comparison of orthostatic tremor activity during standing and walking. Exemplary time-frequency (upper panel) and frequency domain analysis (middle and lower panel) outcomes for two patients (left side: patient 1; right side: patient 5) (**A**,**D**). High-frequency contractions within the gastrocnemius are observable in both patients during the initial stance period. Tremor activity persist during walking but is shifted to a higher frequency range immediately after gait initiation. Tremor frequency returns to the default frequency right after gait termination (**B**,**E**). Changes in tremor intensity during walking show a high inter-individual variability, with an intensity reduction in patient 1 and a profound intensity increase in patient 5 (**C**,**F**). In contrast, tremor coherence (between gastrocnemius and vastus medialis) is only slightly affected by ambulation.

**Figure 2:**
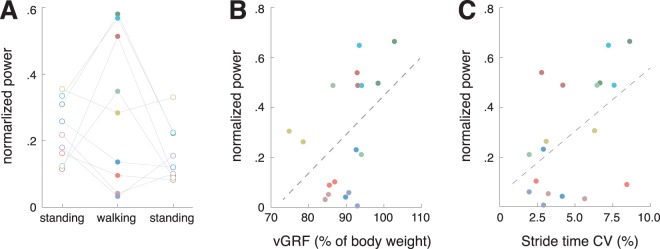
Relation of tremor intensity during walking to gait characteristics. (**A**) Changes in tremor intensity (average of the slow and fast walking trial) during walking compared to standing show a high inter-individual variability. (**B**) On the one hand, tremor intensity is associated to the force and muscle contraction levels applied during stepping. (**C**) On the other hand, high intensity of tremor activity during walking is accompanied by specific gait alterations, namely increased stride time and length variability and a broadened base of support. *vGRF* = *vertical ground reaction force*; *CV* = *coefficient of variation*.

During the initial standing period, all patients exhibited a coherent high-frequency tremor equally in all examined muscles (mean frequency: 15.29 ± 0.17, mean coherence: 0.75 ± 0.02, mean normalized power: 0.23 ± 0.03) and reported the concomitant symptoms of subjective unsteadiness and dizziness. In contrast, no control subject exhibited a coherent high-frequency tremor during standing or walking in the respective muscles (p < 0.001). While walking, all patients reported a relief of symptoms. However, EMG spectral analysis revealed that the tremor persisted during walking but was consistently reset to an even higher frequency (mean frequency: 16.34 ± 0.25; p < 0.001). This effect was equally found for both gait speeds and in all examined muscles. The reset tremor during walking was characterized by a decreased but still high inter-muscular coherence (mean coherence: 0.56 ± 0.03; p < 0.001) (Fig. 1). Changes in tremor intensity during walking showed a high inter-individual variability with an increase in intensity in four patients and a decrease in intensity in the remaining five patients (Fig. 2A).

**Figure 3:**
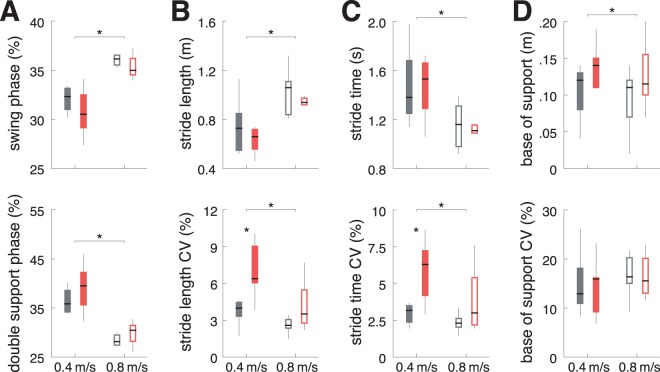
Walking characteristics of patients compared to healthy controls. Gait parameters of patients with OT (red) and healthy controls (gray) at slow (filled boxes) and medium walking speed (open boxes). (**A**) Gait cycle phase parameters, i.e., percentage of swing and double support phase. (**B**) Mean and variability magnitude of stride length. (**C**) Mean and variability magnitude of stride time. (**D**) Mean and variability magnitude of base of support. *Indicates a significant difference. *CV* = *coefficient of variation*.

Correlation analysis revealed that the tremor intensity during gait was highly associated to the patients’ mode of walking, in particular the level of force production and muscle contraction during stepping (vGRF: ρ = 0.295, p = 0.018; aEMG: ρ = 0.677, p < 0.001; Fig. 2B). In turn, higher tremor intensities during walking had a negative impact on walking stability in terms of increased spatiotemporal gait variability (stride length CV: ρ = 0.389, p = 0.002; stride time CV: ρ = 0.357, p = 0.004; Fig. 2C) and a broadened walking base (base of support: ρ = 0.292, p = 0.019). This finding was also reflected in the comparison between gait patterns of patients with OT vs. healthy controls (Fig. 3). Patients walked with an increased spatiotemporal gait variability (stride length CV: p = 0.009; stride time CV: p = 0.003), which was only observable during slow walking and disappeared at faster locomotion. Furthermore, all analyzed gait parameter except base of support CV showed a clear speed dependency for both patients and healthy controls (swing percentage: p < 0.001; double support percentage: p < 0.001; stride length: p < 0.001; stride length CV: p < 0.001; stride time: p < 0.001; stride time CV: p = 0.010; base of support: p = 0.006).

**Figure 4:**
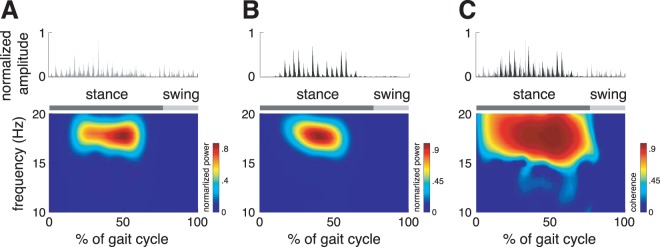
Phase-dependent modulation of tremor frequency within the gait cycle. Rectified EMG-traces and corresponding time-frequency representations (in dependence on the gait cycle phase) of tremor intensity in (**A**) the gastrocnemius and (**B**) the vastus medialis as well as of (**C**) tremor coherence between the two muscles. Tremor activity is phase-dependently modulated during the gait cycle being predominantly present during the stance phase and almost absent during the swing phase of the stride cycle.

Time-dependent tremor analysis within the gait cycle, revealed a persistent but phase-modulated pattern of high-frequency muscles contractions. Accordingly, tremor was predominantly observable during the stance phase (mean peak normalized power: 0.28 ± 0.04; mean peak coherence: 0.70 ± 0.02) but almost completely absent during the swing phase of walking (mean peak normalized power: 0.07 ± 0.01; mean peak coherence: 0.25 ± 0.02; p < 0.001). This effect was equal for both gait speeds and all examined muscles (Fig. 4).

After the walk-stance transition, the coherent tremor was found to be reset to the initially lower frequency (mean frequency: 15.35 ± 0.17 Hz, mean coherence 0.71 ± 0.02). However, the tremor showed a variable latency to reach the mean intensity level of the initial standing period (mean onset latency: 3.7 ± 0.9 s, range 0.1–37.9 s), resulting in a slightly decreased average intensity of the second stance period (mean normalized power: 0.16 ± 0.03). This effect was again equal for both gait speeds and all examined muscles. All of the observed effects were independent of the status of medication of patients.

## Discussion

In the present study, we studied tremor activity during stance-walk transitions and continuous walking as well as gait performance in patients with OT. The main three findings were: (1) coherent high-frequency tremor bursts persist during walking but are shifted to a higher frequency range; (2) tremor intensity during walking is phase-dependently modulated and scales with the leg force levels applied during stepping; (3) intense tremor activity during walking is associated to specific gait alterations. In the following, these observations will be discussed with respect to their implications for the pathomechanism and the gait disorder in OT.

Although all patients consistently reported a relief of unsteadiness when starting to ambulate, tremor activity was found to persist while walking in accordance to previous reports^7,8^. The high inter-muscular coherence of tremor bursts during ambulation indicates that the tremor pattern is still governed by a supraspinal oscillatory source. However, immediately after gait initiation, the frequency of coherent muscle contractions was consistently shifted to a higher frequency range (average increase of 1.2 ± 0.1 Hz) compared to tremor frequency while standing. Tremor frequency shifts of similar order have been observed in other central tremor forms as Parkinson’s disease or essential tremor in the presence of rhythmically paced voluntary limb movements^14^. In these cases, it was assumed that the modulation in tremor frequency might result from a nonlinear interaction between the tremor oscillator and the rhythmically paced motor commands. Accordingly, the tremor resetting during walking in OT could result from an interference of the default tremor pattern with either proprioceptive afferent feedback, spinal or supraspinal locomotor commands. The first two possibilities seem unlikely, as both peripheral nerve stimulation as well as spinal cord stimulation were found to not influence OT rhythm^7,17–19^. There is however evidence in favor of the third alternative in that OT frequency was found to be transiently reset by electrical stimulation over the posterior fossa^17^. Supraspinal locomotor areas in the cerebellum, thalamus, and motor cortex have a profound overlap with the ponto-cerebello-thalamo-cortical tremor network that was recently described in OT^4,5,20^. In particular cerebellar locomotor areas are known to exhibit oscillatory pacemaker activity for speed regulation during walking^21,22^. An interference of rhythmic activity in these areas with the default tremor activity appears therefore most likely to underlie the observed frequency modulation during walking in patients with OT.

Changes in tremor intensity during walking compared to standing showed a high inter-individual variability and proportionally scaled with leg muscle contraction intensity and the effective force levels applied during walking. Furthermore, tremor intensity during walking was intra-individually modulated in dependence on the gait cycle, being most prominent during the stance phase (i.e., in the presence of muscle contractions under load) and almost absent during the swing phase, when the leg was lifted off the floor. Taken together, these observations indicate that the central oscillatory pattern that appears to be active during symptomatic (i.e., standing) as well as non-symptomatic states (i.e., lying)^4^ only manifests peripherally in muscles being contracted under load, i.e., during activation of Golgi tendon organ (GTO) afferents that directly project to Ib interneurons^23^. This in turn would suggest that the peripheral manifestation of OT does not occur via a direct projection of central oscillatory sources to spinal motoneurons, but is rather mediated via spinal interneurons that signal the loading state of respective muscles^17^.

Intense OT activity during walking was further associated with specific gait alterations, namely a broadened base of support and high spatiotemporal gait fluctuations that were pathologically increased in particular during slow walking when compared to healthy controls. Both of these gait alterations are linked to an impaired regulation of dynamic gait stability and indicate an increased risk of falling^24–28^. Moreover, these gait impairments resemble the phenotype of either a cerebellar or a sensory ataxic gait, which are both characterized by a staggering wide based gait pattern especially at slow walking modes^27,29,30^. Signs for mild cerebellar motor abnormalities and a reduced vibration sense of the feet were frequently observed in our patients, in accordance to former studies^4,31^. Furthermore, previous evidence supports both either a cerebellar or a peripheral sensory deficit underlying the gait disorder in OT. On the one hand, recent studies suggest OT to be a cerebellar pathology indicated by changes in cerebellar grey matter volume and alterations in the functional connectivity between the cerebellum and the supplementary motor area that both correlated with tremor and clinical severity in patients with OT^32–34^. There is further evidence for alterations in the connectivity of fronto-cerebellar circuits in OT that are linked to deficits of specific neuropsychological functions observed in patients^35,36^. Moreover, repetitive transcranial magnetic stimulation of the cerebellum was demonstrated to reduce tremor severity and associated changes in functional brain connectivity of patients with OT^32^. On the other hand, Fung *et al*. provided evidence for an impairment of peripheral sensory feedback underlying the symptoms emergence in OT^13^. Accordingly, it was suggested that during prolonged standing in OT, proprioceptive feedback from the periphery becomes increasingly synchronized at the tremor frequency. This tremor-locking of proprioceptive afferents would disrupt normal peripheral feedback regulation of posture and give rise to an increased co-contraction of anti-gravity musculature leading to a vicious cycle of escalating subjective and objective postural unsteadiness^13,19^. This assumption was further supported by a positive Romberg’s sign, i.e., an aggravated balance disequilibrium in patients with OT while standing with eyes closed^13^. Thus, disturbed peripheral feedback during walking might hinder patients with OT to adequately adjust their stride-to-stride pattern for unintended irregularities^37,38^, leading to the observed staggering wide base gait pattern. However, proprioceptive feedback available during repetitive tremor-free episodes (i.e., swing phases), might be sufficient to continuously update dynamic limb positions during walking and prevent patients with OT from becoming subjectively unsteady while ambulating.

As most studies on OT, the present findings are limited by the relatively small sample size due to the rarity of the disease. Moreover, walking on treadmill cannot be directly compared to overground walking performance. Previous studies however, demonstrated that in particular the magnitude of spatiotemporal gait fluctuations during treadmill vs. overground walking is comparable in healthy subjects and patients with neurological gait disorders^30,39^. Future studies on a larger sample size of patients with OT including the assessment of overground walking performance are required to confirm the findings of this study.

In conclusion, OT during walking was found to persist while resetting of tremor frequency indicates a nonlinear interference between the default OT rhythm with oscillatory supraspinal locomotor activity. OT intensity was shown to be phase-dependently modulated during the gait cycle and linked to the loading state of respective muscles. Thus, peripheral manifestation of OT seems to be mediated via spinal interneurons signaling muscle loading. Finally, OT during ambulation was linked to specific gait alterations that suggest an underlying cerebellar and/or peripheral sensory deficit.

## Data Availability

The datasets generated and analyzed during the current study are available from the corresponding author on reasonable request.
